# Self-Generated Buried Submicrocavities for High-Performance Near-Infrared Perovskite Light-Emitting Diode

**DOI:** 10.1007/s40820-023-01097-3

**Published:** 2023-05-15

**Authors:** Jiong Li, Chenghao Duan, Qianpeng Zhang, Chang Chen, Qiaoyun Wen, Minchao Qin, Christopher C. S. Chan, Shibing Zou, Jianwu Wei, Zuo Xiao, Chuantian Zuo, Xinhui Lu, Kam Sing Wong, Zhiyong Fan, Keyou Yan

**Affiliations:** 1 School of Environment and Energy, State Key Lab of Luminescent Materials and Devices, Guangdong Provincial Key Laboratory of Solid Wastes Pollution Control and Recycling, South China University of Technology, Guangzhou, 510000 People’s Republic of China; 2 Department of Electronic and Computer Engineering, The Hong Kong University of Science and Technology, Clear Water Bay, Kowloon, Hong Kong People’s Republic of China; 3 Department of Physics, The Chinese University of Hong Kong, Shatin, Hong Kong People’s Republic of China; 4 Department of Physics, The Hong Kong University of Science and Technology, Clear Water Bay, Kowloon, 999077 Hong Kong People’s Republic of China; 5 School of Chemistry and Chemical Engineering, Guangxi University, Nanning, 530004 People’s Republic of China; 6 Center for Excellence in Nanoscience (CAS), Key Laboratory of Nanosystem and Hierarchical Fabrication (CAS), National Center for Nanoscience and Technology, Beijing, 100190 People’s Republic of China

**Keywords:** Perovskite light-emitting diodes, Downward recrystallization, Buried submicrocavities, Light out-coupling efficiency, Radiative recombination

## Abstract

Embedding submicrocavities is an effective approach to improve the light out-coupling efficiency (LOCE) for planar perovskite light-emitting diodes (PeLEDs). In this work, we employ phenethylammonium iodide (PEAI) to trigger the Ostwald ripening for the downward recrystallization of perovskite, resulting in spontaneous formation of buried submicrocavities as light output coupler. The simulation suggests the buried submicrocavities can improve the LOCE from 26.8 to 36.2% for near-infrared light. Therefore, PeLED yields peak external quantum efficiency (EQE) increasing from 17.3% at current density of 114 mA cm^−2^ to 25.5% at current density of 109 mA cm^−2^ and a radiance increasing from 109 to 487 W sr^−1^ m^−2^ with low rolling-off. The turn-on voltage decreased from 1.25 to 1.15 V at 0.1 W sr^−1^ m^−2^. Besides, downward recrystallization process slightly reduces the trap density from 8.90 × 10^15^ to 7.27 × 10^15^ cm^−3^. This work provides a self-assembly method to integrate buried output coupler for boosting the performance of PeLEDs.

**Supplementary Information:**

The online version contains supplementary material available at 10.1007/s40820-023-01097-3.

## Highlights

- Synergistic effect triggers the Ostwald ripening for the downward recrystallization of perovskite to form buried submicrocavities as light output coupler.
- The simulation suggests the buried submicrocavities can improve the light out-coupling efficiency from 26.8% to 36.2% for near-infrared light.
- Light-emitting diodes yields peak external quantum efficiency increasing from 17.3% at current density of 114 mA cm^−2^ to 25.5% at current density of 109 mA cm^−2^ and a radiance increasing from 109 to 487 W sr^−1^ m^−2^ with low rolling-off.

**Supplementary Information:**

The online version contains supplementary material available at 10.1007/s40820-023-01097-3.

## Graphical abstract

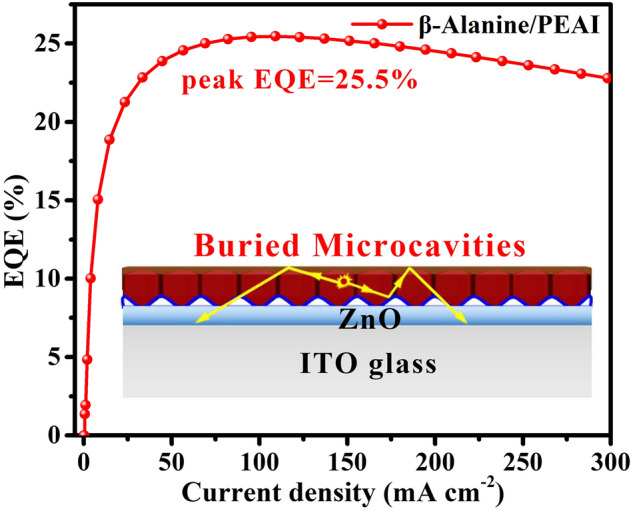

## Introduction

With high photoluminescence quantum yield (PLQY), tunable emission wavelength and narrow full width at half maximum (FWHM), perovskite light-emitting diodes (PeLEDs) are regarded as the promising candidate for next-generation solid-state lightings and high-definition displays [1–5]. The external quantum efficiency (EQE) of PeLEDs is, however, restricted by factors such as the quality of the perovskite films, probability of balanced charge injection and light out-coupling efficiency (LOCE) [6–8].

At present, recrystallization has become one of the commonly used methods to reduce defects, suppress nonradiative recombination and optimize the quality of perovskite films, including post-processing, vapor-assisted annealing and flash-induced annealing [9–11]. In addition, the LOCE also plays a crucial role in the electroluminescence (EL) [12, 13]. Theoretical analysis shows that the LOCE of planar PeLEDs is ~ 20%, which is mainly due to the high refractive index of the perovskite layer, trapping most of the light inside [14–17]. Therefore, it is still meaningful to develop a simple optical output micro-nanostructure to effectively improve the LOCE and performance of PeLEDs.

Herein, we adopted a simple route to post-treat perovskite film with phenethylammonium iodide (PEAI) and triggered downward recrystallization of perovskite, resulting in spontaneous formation of buried submicrocavities. The simulation result suggests the buried submicrocavities can improve the LOCE from 26.8 to 36.2% by coupling the waveguide modes to substrate modes. Besides, Ostwald ripening process slightly passivates the traps and thus reduces the nonradiative recombination losses as well. Therefore, PeLED yields peak EQE increasing from 17.3% at current density of 114 mA cm^−2^ to 25.5% at current density of 109 mA cm^−2^ and a radiance increasing from 109 to 487 W sr^−1^ m^−2^ with low rolling-off. The turn-on voltage at 0.1 W sr^−1^ m^−2^ is 1.15 V, lower than 1.25 V for the control. The statistical result indicates 95% of the devices with PEAI can achieve a peak EQE of more than 20%, with nice repeatability PeLED exhibits good spectral stability under different bias voltages and the *T*_75_ (*T*_75_, defined as the time taken for the EQE to drop to 75% of its initial value) exceeds 15 h.

## Material and Methods

### Materials

The zinc oxide nanoparticles (ZnO NPs) were synthesized from potassium hydroxide (KOH, CAS no. 1310-58-3, Aladdin, 99.99%) and zinc acetate dihydrate (Zn(Ac)_2_^**.**^2H_2_O, CAS no. 5970-45-6, Macklin, 99.99%). 2,2′,7,7′-Tetrakis [*N*, *N*-di(4-methoxyphenyl) amino]-9,9′-spirobifluorene (Spiro-OMeTAD, CAS no. 207739-72-8, 99.95%), Phenethylammonium iodide (PEAI, CAS no. 151059-43-7, ≥ 99.5%) and Lead iodide (PbI_2_, CAS no. 10101-63-0, 99.99%) were purchased from Xi’an Polymer Light Technology Corp. *N*,*N*-Dimethylformamide (DMF, CAS no. 68-12-2, 99.5%), *β-*Alanine (CAS no. 107-95-9, 99%) and Molybdenum trioxide (MoO_x_, CAS no. 1313-27-5, 99.95%) were purchased from Aldrich. Formamidinium iodide (FAI, CAS no. 879643-71-7, 99.5%) and the patterned ITO glass was purchased from Advanced Election Technology Co., Ltd.

### Synthesis of ZnO NPs

ZnO nanoparticles were synthesized by reacting KOH with Zn(Ac)_2_·2H_2_O in methanol for 2 h at 60 °C. After centrifuging and dispersing in methanol for three times, the precipitate is dispersed it in methanol and chloroform for using.

### Device Fabrication

To prepare a perovskite precursor solution with a concentration of 0.7 M, *β-*Alanine, FAI, PbI_2_ were dissolved in a molar ratio of (0, 0.1, 0.15, 0.2): 1.8: 1.0 in 1 mL of DMF and stirred overnight at 60 °C. After cleaning with cleaning agent, deionized water, acetone, isopropanol, and absolute ethanol, the patterned ITO glass (sheet resistance of 15 Ω sq^−1^) was dried with nitrogen, and then the substrate was further cleaned with UV-Ozone. The substrate was spin-coated with 6 mg mL^−1^ ZnO NPs as electron transport layer at 4000 rpm for 30 s, and annealed at 150 °C for 30 min in a fume hood. The perovskite precursor solution was spin-coated on the previous substrate at 6000 rpm for 20 s under nitrogen atmosphere and annealed at 150 °C for 60 s. Chlorobenzene was dripped as an antisolvent at 5 s. PEAI (6 mg mL^−1^) was spin-coated for 30 s at 4,000 rpm at room temperature, then annealed at 75 °C for 10 min. After that, Spiro-OMeTAD (24 mg mL^−1^) was spin-coated on the perovskite film as hole transport layer. Using vacuum evaporation equipment, 5 nm MoO_x_ and 100 nm Ag electrode were sequentially deposited, whereby the effective area of the device (the overlapping area of the ITO electrode and the Ag electrode) is 8 mm^2^.

### Film Characterization

Verify crystallinity of perovskite films with an X-ray diffractometer (XRD) (Model: Empyrean), measure surface roughness of perovskite films with an Atomic Force Microscope (AFM) (Model: MFP-3D-S), and characterize surface morphology of perovskite films with an ultra-high resolution field emission scanning electron microscope (SEM) (Model: Merlin). An ultraviolet visible (UV) spectrophotometer (Model: UV-2600) is used to measure absorbance values of ZnO NPs and perovskite films. The energy levels of perovskite films are measured by UV photoelectron spectroscopy (UPS). The Fourier Transform Infrared Spectrometer (FTIR Spectrometer) (Model: CCR-1) was used to measure the changes in characteristic peaks of functional groups. A 590 nm laser, a flame spectrometer, and an integrating sphere were also used to measure the photoluminescence of perovskite films.

### Time-Resolved Photoluminescence Measurements

Excitation with 635 nm pulse laser (Edinburgh instruments) at 200 kHz focused on an encapsulated sample at an excitation power of 0.5 μW (~ 8 nJ cm^−2^). The PL was collected at ~ 815 nm with a spectrometer onto a photon counter to carry out time correlated single photon counting measurements. The longest lifetime samples had decays which were longer than the repetition rate of the laser (background is subtracted), we are limited by our system. The fittings are performed with biexponentials, with y_0_ offset set to 0 for all cases.

### Performance and Stability Measurement

A commercialized system (XPQY-EQE, Guangzhou Xi Pu Optoelectronics Technology Co., Ltd.) equipped with an integrated sphere (GPS-4P-SL, Labsphere) and a photodetector array (S7031-1006, Hamamatsu Photonics) was used to analyze the lifetime, EL spectra and EQE of the PeLEDs. The test parameters were set as follows: area: 0.08 cm^−2^; target wavelength: 810 nm; bias voltage: 0–4 V; rate: 0.05 V s^−1^. An SS-F5-3A solar simulator (Enli Technology Co., Ltd.) was used to measure the current density–voltage (*J-V*) characteristics of the PV performance in the glove box using a computer-controlled Keithley 2400 sourcemeter under a simulated AM 1.5 G solar illumination (100 mW cm^−2^).

### Optical Simulation

The optical simulation was performed with the Lumerical FDTD package. Dipole sources with in-plane and out-of-plane polarizations were utilized for light out-coupling calculations. Material index information: Spiro-OMeTAD (*n = *1.63), FAPbI_3_ (n ~ 2.40, 809 nm), ZnO (*n = *1.96), MoOx (*n = *2.05), Ag index from Palik (0–2 m$\upmu$) included in Lumerical FDTD package.

## Results and Discussion

### Intermolecular Synergistic Effect for Downward Recrystallization

As well known, the defects in the emission layer that jeopardize the radiative recombination are extremely detrimental for fabricating high-performance PeLEDs. There are two approaches to enhance light-emitting performance. First, 2D/3D perovskite with multiple quantum wells was engineered to funnel energy and screen the defect for the radiative recombination, and the 2D components of which can also stabilize the intrinsic structure and radiative centers [18–20]. Second, a series of ligands have been added into precursor to passivate perovskite defects and engineer the micro-nanostructure for high-performance PeLED [21–23]. For example, 5-Aminovaleric acid (5AVA) was introduced into the precursor solution of FAPbI_3_ to induce the sub-micron scale islands to effectively extract trapped light in planar PeLEDs [21]. The structure-performance relationship was illustrated by the identification of molecular length, heteroatoms (C–O–C), and functional groups (amino and carboxyl groups). Amino group (NH_2_) can interact with FAI to induce vertical structure and carboxyl group (COOH) fills the halide vacancy defects [4]. Heteroatoms (C–O–C) polarizes the passivation amino group and enhances coordination of the functional groups with the perovskite defect site [23]. Therefore, it is meaningful to study the synergistic effect between amino acids and 2D components.

**Fig. 1:**
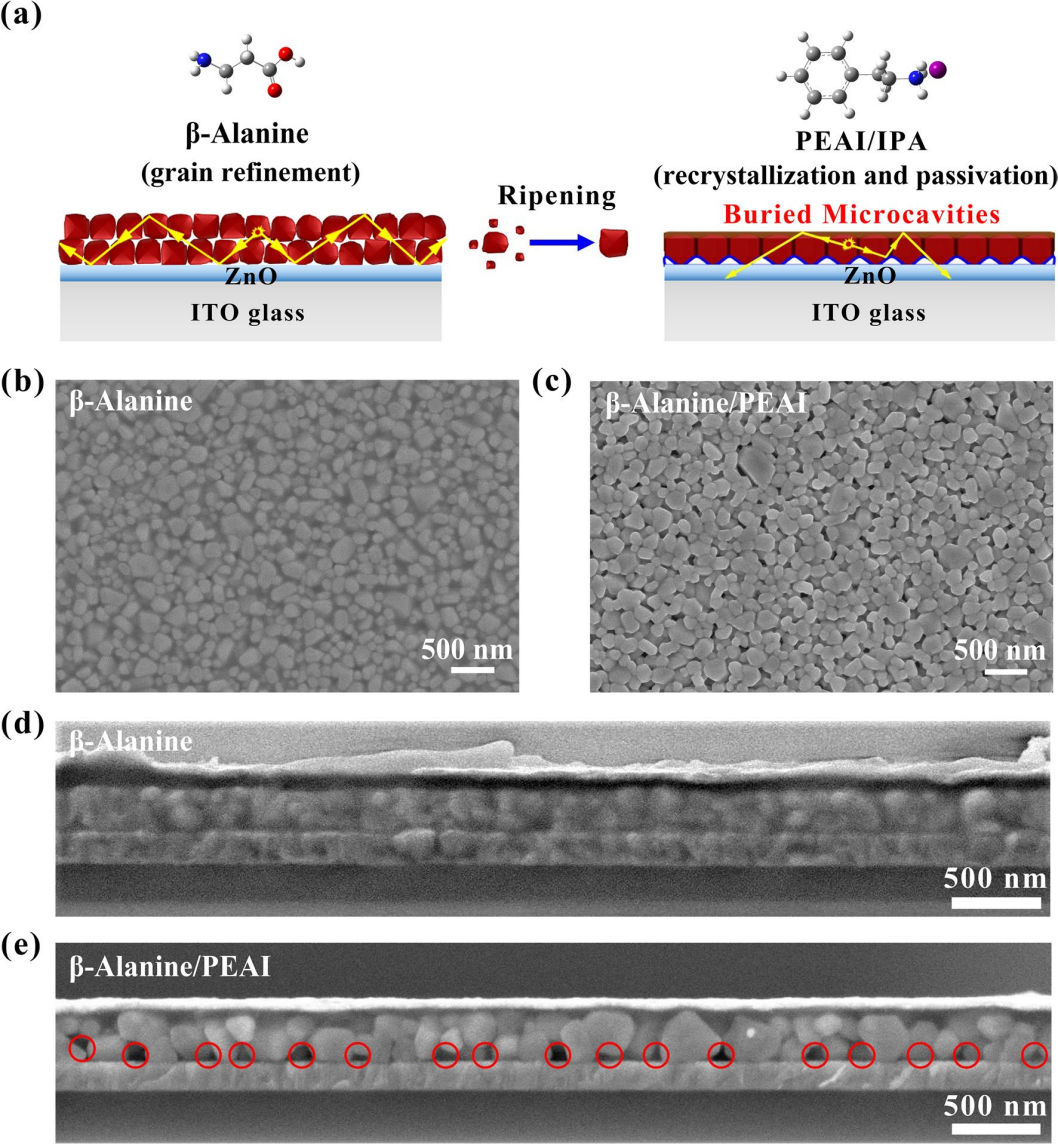
**a** Schematic diagram of downward recrystallization for buried submicrocavities to change light pathways. SEM images of perovskite films with **b** *β-*Alanine and **c** *β-*Alanine/PEAI. The cross-section SEM images of PeLEDs with **d** *β-*Alanine and **e** *β-*Alanine/PEAI

We found that the *β-*Alanine works synergistically with the subsequent PEAI post-treatment for high performance. Figure 1a is the schematic diagram of the recrystallization. *β-*Alanine can shape the grain with more uniform and nanosized (Fig. 1b) without the convex-concave defects (Fig. S1). PEAI post-treatment induces the Ostwald ripening (Fig. 1a, right) and the recrystallization can be seen from the morphology change (Fig. 1c). PEAI post-treatment was also conducted on perovskite films with 5AVA (Fig. S2). Unlike *β-*Alanine, there is no significant synergistic effect on the morphology probably due to the steric effects. As shown in Fig. S3, *β-*Alanine-based perovskite films were post-treated with PEAI, and due to the weak steric hindrance effect, PEAI can induce Ostwald ripening on perovskite surface and cause recrystallization of perovskite grains. However, when PEAI post-treated the 5AVA-based perovskite films, due to the strong steric hindrance effect, the interaction between PEAI and perovskite surface was blocked to a great extent [24].

The phase change may trigger the recrystallization. After PEAI layer was spin-coated on the perovskite film, XRD patterns of δ-FAPbI_3_ phase at 21.6° weakened and the transformation of the α-FAPbI_3_ phase can be judged from the stronger peaks at 14.0° and 28.0° (Fig. S4a) [25]. Hence, it confirms that PEAI migrates to assist δ-FAPbI_3_ recrystallization into α-FAPbI_3_. Surprisingly, after PEAI post-treatment, no diffraction peak of PEA_2_PbI_4_ (0 0 2) appeared at 5.1°, but a new diffraction peak appeared at 7.3° (Fig. S4b). These results demonstrated that FA^+^ is easily extracted by IPA solution and then PEA^+^ cation enters into the [PbI_6_]^4−^ inorganic framework and forms an intermediate (newly appeared XRD peak at 7.3°) [26]. Meanwhile, we performed grazing incidence wide-angle X-ray scattering (GIWAXS) measurement to focus on the surface phase change of perovskite films (Fig. S5). Although 2D GIWAXS suggests that the pristine, *β-*Alanine, and *β-*Alanine/PEAI-based perovskite films all show characteristic *α*-phase scattering ring at |*q*|= 1.00 Å^−1^, *δ*-phase scattering ring appearing at |*q*|= 0.8 Å^−1^ gradually weakens in the sequence of pristine, *β-*Alanine, and *β-*Alanine/PEAI samples, where the scattering ring of GIWAXS at |*q*|= 0.8 Å^−1^ corresponds to the diffraction peak of XRD at 21.6° (0 3 3) [27–29]. Therefore, one can infer that the phase change starts from top to bottom.

The downward recrystallization is also confirmed by cross-section SEM. The downward growth induces Ostwald ripening to swallow small grains (Fig. 1a) and yields submicrocavities in the buried film (Fig. 1d, e). The originally closely-arranged perovskite grains intermittently ripen to form large grains and induce irregular submicrocavities. The buried interface of the submicrocavities can redirect the direction of light propagation of the in-plane guided mode in the perovskite layer and transcend the captured photons, thus facilitating light extraction (Fig. 1a light pathways) [30].

Under recrystallization, surface properties changed. The roughness of the perovskite films also affects the deposition of the upper layer to a certain extent, so we tested the surface roughness of the pristine, *β-*Alanine, and *β-*Alanine/PEAI-based perovskite films by atomic force microscopy (AFM) (Fig. S6) [31, 32]. The root mean square (RMS) of the perovskite film after addition of *β-*Alanine only changed negligibly from 18.7 to 19.2 nm. Nevertheless, the post-treatment of PEAI lessens the RMS to 16.1 nm, indicating that PEAI is easier to deposit on the grain boundaries, reducing the height difference between the grain surfaces and the grain boundaries, creating favorable conditions for the deposition of hole transport materials [33]. In addition, the water contact angle measurement of perovskite film was also shown in Fig. S7. Due to the excellent solubility of *β-*Alanine in water, the water contacts angle of perovskite film after adding *β-*Alanine decreased from 35.9° to 13.6°. However, after PEAI passivation, the water contacts angle of perovskite film increased to 48.8°, which is very beneficial to the humidity stability of PeLEDs.

The interaction between PEAI and perovskite film was inferred by X-ray photoelectron spectroscopy (XPS). As shown in Fig. S8, after PEAI post-treatment, the Pb *4f* peak and I *3d* peak shifted towards the direction of low binding energy, which was attributed to the increased electron cloud density around the lead due to the interaction between PEAI and Pb^2+^ [34, 35]. Furthermore, Fourier transform infrared spectroscopy (FTIR) measurements were performed on perovskite films. The N–H stretching vibration peak of the *β-*Alanine/PEAI-based perovskite film starts to shift to high wavenumbers, which further proves the strong interaction between PEAI and perovskite film (Fig. S9) [21, 23, 36]. The stretching vibration peak at 1535 cm^−1^ wavenumber is the benzene ring skeleton peak introduced by PEAI post-treatment [37].

### LOCE Improvement and Insight

**Fig. 2:**
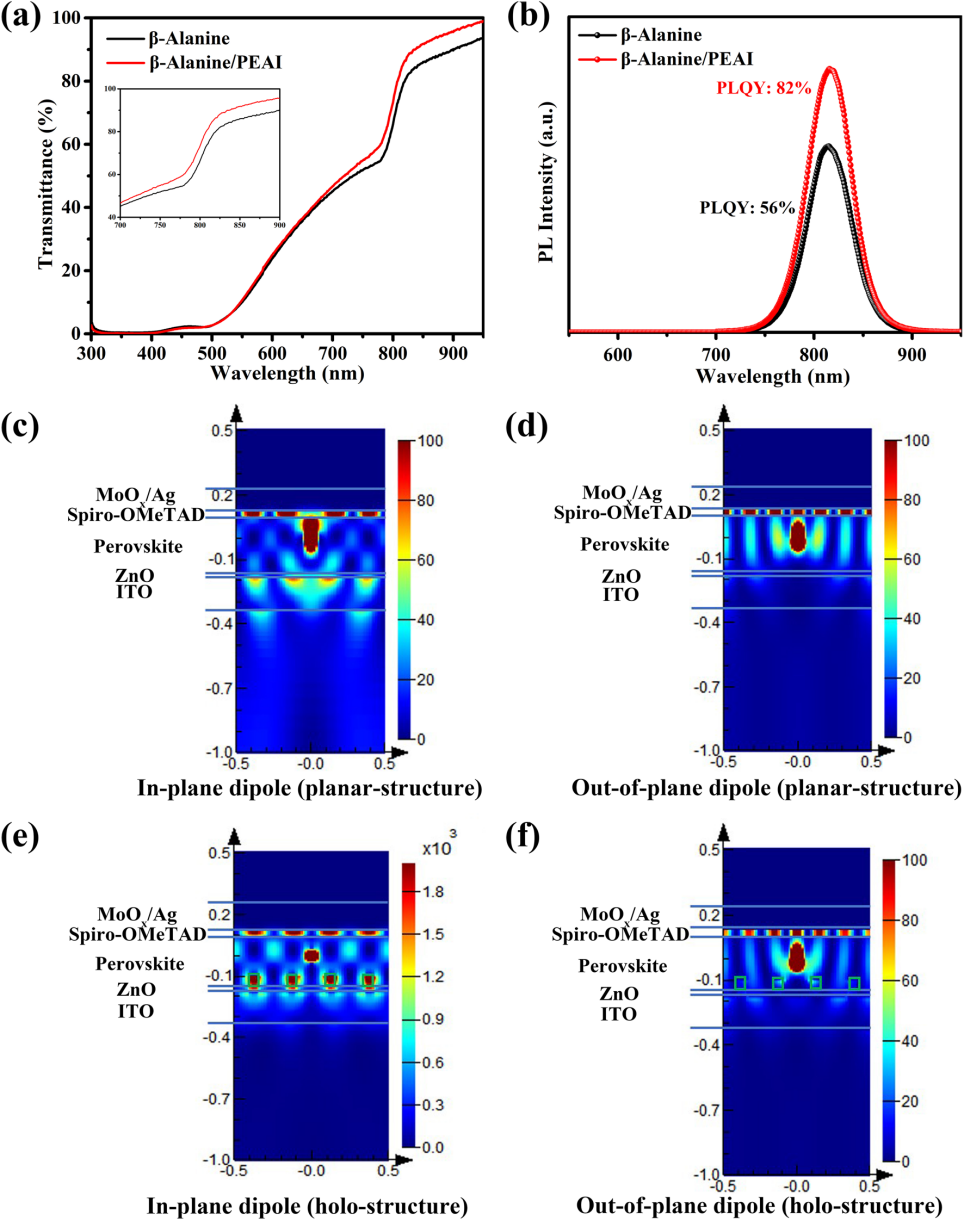
**a** Transmittance spectra of perovskite films. **b** PL spectra. E^2^ (V/m)^2^ intensity profiles for **c** In-plane dipole (planar structure). **d** Out-of-plane dipole (planar structure). **e** In-plane dipole (holo-structure). **f** Out-of-plane dipole (holo-structure). The unit for color bar is (V/m)^2^. Light propagates downwards

We measured the transmittance and PLQY to evaluate the light-emitting loss. Figure 2a shows the optical transmission of the perovskite films. As the thickness reaches 270 nm, the incident light can be absorbed by perovskite films to a large extent in the visible wavelength range. However, in the near-infrared region, the light transmission of perovskite film after recrystallization is significantly enhanced, which further confirms the existence of submicrocavities. As shown in Fig. 2b, the optimal *β-*Alanine/PEAI-based perovskite film exhibited notably enhanced PL emission (with a PLQY of 82%) compared to *β-*Alanine-based perovskite film (with a PLQY of 56%), indicating enhanced radiative recombination by recrystallization.

To evaluate the improvement of LOCE by submicrocavities, we studied the optical loss through theoretical simulation. During the simulation, we assumed that the dipoles move equally and randomly along the *X*, *Y*, and *Z* directions, and that the lateral dimension of the layer is infinite and the glass substrate is also considered to be semi-infinitely long along the *Z* direction. The thicknesses of Ag, MoO_*x*_, Spiro-OMeTAD, Perovskite, ZnO, and ITO are taken as 100, 3, 30, 270, 10, and 150 nm, respectively, and the refractive indices of MoO_*x*_, Spiro-OMeTAD, Perovskite, and ZnO are 2.05, 1.63, 2.40, and 1.96 (Fig. S10a) [38–40]. At the same time, the average size of the hole caused by recrystallization is about 70 nm × 70 nm, and the hole interval is about 180 nm (Fig. S10b).

Figure 2c, d showes the simulation results. Through calculations, we obtained that in the planar structure, the LOCEs of the in-plane and out-of-plane dipoles are 36% and 8.4%, respectively, and the final LOCE is 26.8%. From the simulation results in Fig. 2e, f, for the holo-structure like the experiment with submicrocavities, the LOCEs of the in-plane and out-of-plane dipoles are 52.9% and 2.9%, respectively, and the combined LOCE is 36.2%. Therefore, after the recrystallization after PEAI, the LOCE is possibly increased from 26.8 to 36.2%, which has great potential to improve the EQE of the device.

### Device Performance

**Fig. 3:**
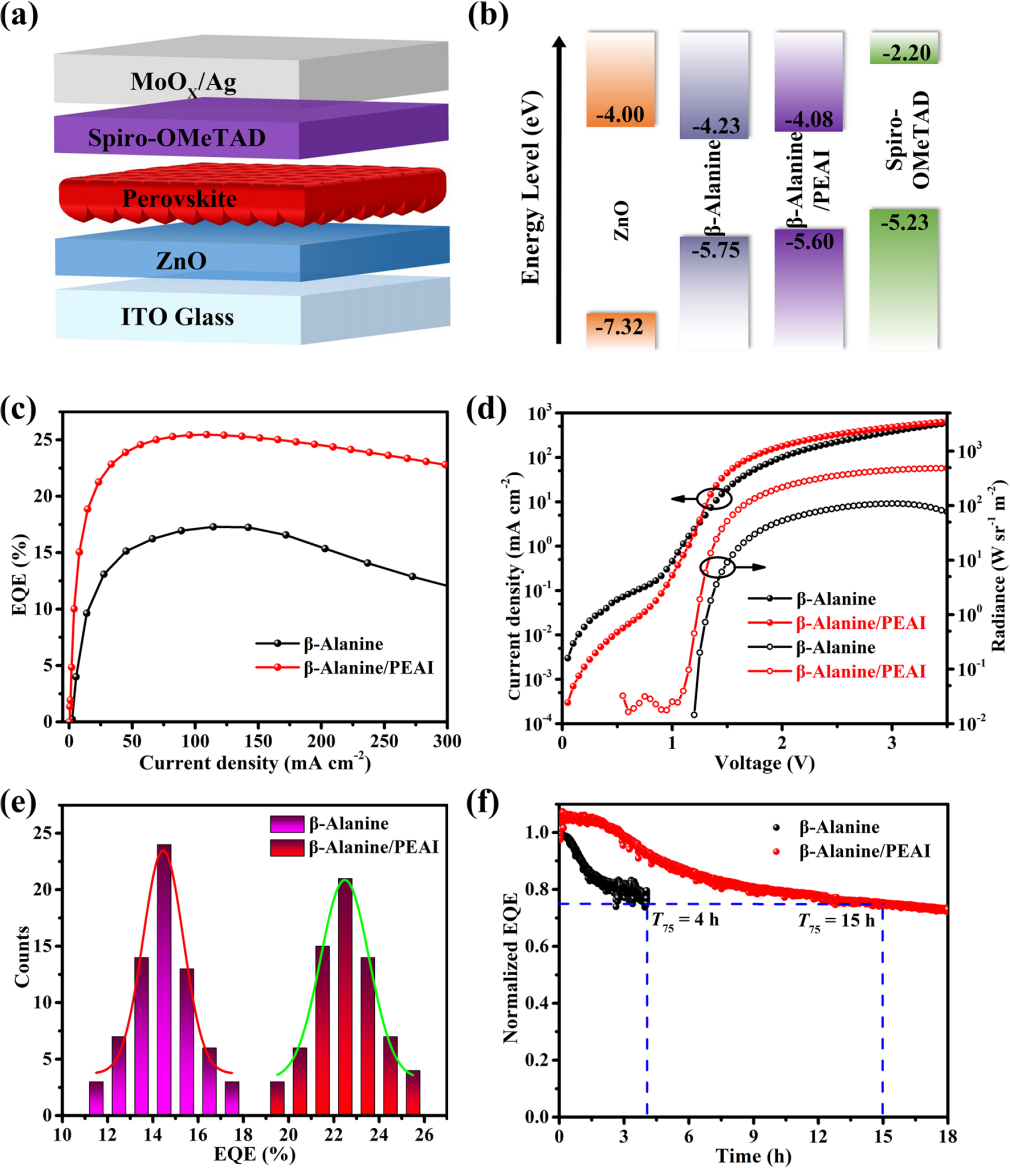
**a** Device structure. **b** Energy level diagram. **c** EQE diagrams. **d** Current density–voltage-radiance curves. **e** Efficiency statistics histograms. **f** Device lifetime plots at a current density of 62.5 mA cm^−2^

To evaluate the performance, we fabricated the PeLEDs with a structure of ITO/ZnO/Perovskite/Spiro-OMeTAD/MoO_*x*_/Ag (Fig. 3a). In terms of energy level alignment, the effective injection of electrons and holes is vital for the realization of high-performance devices, which requires the matching of energy levels of perovskite layer and transport layers. Figure 3b is the energy level diagram of the device based on the test results of the literature and ultraviolet photoelectron spectroscopy measurements (UPS) (Fig. S11) [41]. Compared with the valence band (VB) of *β-*Alanine-based perovskite film (− 5.75 eV), there is a lower energy level barrier between the VB of *β-*Alanine/PEAI-based perovskite film (− 5.60 eV) and the highest occupied molecular orbital (HOMO) of Spiro-OMeTAD (− 5.23 eV), which is conducive to reducing the energy loss of PeLEDs [42, 43].

The light-emitting metrics is discussed in the following. The device without *β-*Alanine and PEAI only has peak EQE of 6.8% with a radiance of 23 W sr^−1^ m^−2^ and rolling-off rapidly, which is far away from reaching commercial requirements (Fig. S12). As shown in Fig. 3c, with the addition of *β-*Alanine in the precursor solution, the peak EQE of PeLED reached 17.3% at current density of 114 mA cm^−2^, which undoubtedly shows that the addition of *β-*Alanine improves radiative recombination and reduces the defects of perovskite layer. Furthermore, a record breaking EQE of 25.5% with low rolling-off was measured at a current density of 109 mA cm^−2^ after PEAI post-treatment. In addition, the radiance was increased from 109 to 487 W sr^−1^ m^−2^, and the leakage current was also suppressed (Fig. 3d).

It is worthy of affirmation that the device can still maintain a high radiance under the premise of ensuring high electro-optical conversion efficiency. As shown in Figs. S13 and S14, the PeLED with PEAI post-treatment has a stronger EL spectrum at 807 nm, and exhibits good spectral stability under different bias voltages. Under the external voltage, the EL spectrum of *β-*Alanine/PEAI-based PeLED was blue shifted from 809 nm at 1.5 V to 805 nm at 3.5 V. The slight shift of the band-edge as a function of injected carrier density is due to band filling with large injection current. Charge accumulation in the perovskite films leads to an increase in the intrinsic bandgap that follows the Burstein-Moss band filling model, which has been studied in previous work [44]. In the statistics of 140 devices with PEAI post-treatment, 95% of the devices can achieve the peak EQE of more than 20%, indicating that the device performance has excellent repeatability (Fig. 3e). In addition, the *β-*Alanine/PEAI-based device exhibits a *T*_75_ of 15 h (Fig. 3f). We conducted ToF–SIMS tests to explore whether halide diffusion exists in this experiment, thus affecting the stability of the device. As shown in Fig. S15, we explored ToF–SIMS of iodide-anion distribution depth profiles were explored before and after *β-*Alanine/PEAI-based PeLED test. It can be seen that iodide ions in the perovskite layer in device diffuse to the interface between the Ag electrode and the Spiro-OMeTAD. Therefore, even though there is halide diffusion, there is not significant adverse effect on the top electrode under inert atmosphere, which is the key to achieve acceptable stability of our device. This good stability can be attributed partially to the reduction in defects in recrystallized perovskite film caused by PEAI post-treatment.

As shown in Fig. S16, even if a small amount of *β-*Alanine is added into precursor solution, the EQE of the device can be improved and the rolling-off can be alleviated. However, when the concentration of *β-*Alanine is superfluous (*n*_*β-*Alanine_: *n*_Pb_ = 0.2: 1.0), the EQE of device gradually decreases. Meanwhile, the dependence of device performance on PEAI concentration indicates that EQE of device with high PEAI concentration (8 mg mL^−1^) begins to decline, possibly because the thicker PEAI layer inhibits carrier transport and reduces radiative recombination (Fig. S17).

### Device Mechanisms

**Fig. 4:**
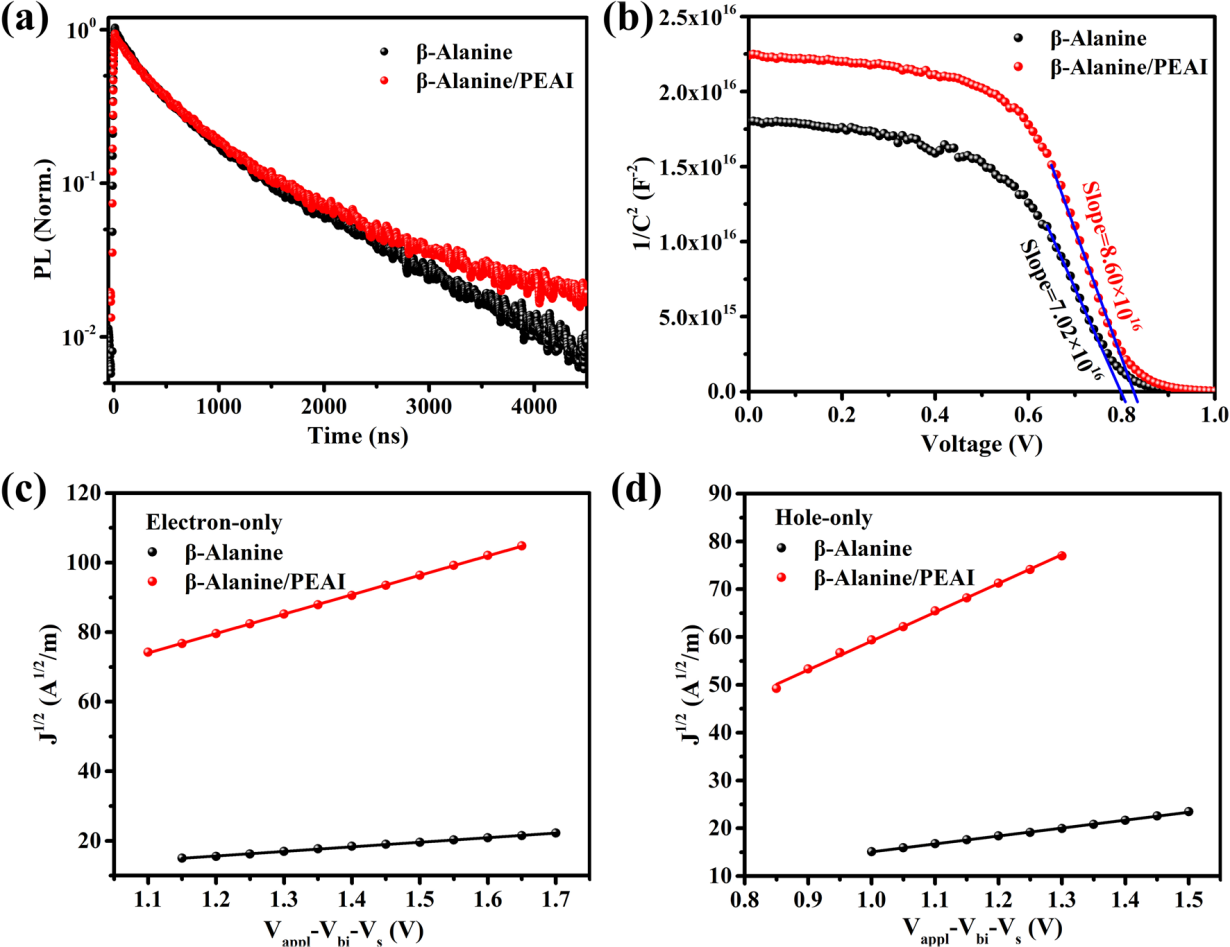
**a** TRPL spectra of perovskite films. **b** Mott-Schottky plots. **c** *J*^1/2^–*V*_appl_–*V*_bi_–*V*_s_ curves of electron-only devices with a structure of ITO/ZnO/Perovskite/PCBM/Ag. **d** *J*^1/2^–*V*_appl_–*V*_bi_–*V*_s_ curves of hole-only devices with a structure of ITO/PEDOT: PSS/Perovskite/Spiro-OMeTAD/Ag

To explore the effect of recrystallization on the charge transfer kinetics, the carrier lifetime of the perovskite films was measured by time-resolved photoluminescence (TRPL) (Fig. 4a). TRPL spectra showed that the exciton lifetime (1083 ns) of *β-*Alanine/PEAI-based perovskite film is longer than that of *β-*Alanine-based perovskite film (896 ns), suggesting that the PEAI layer plays a positive role in inhibiting the defects of perovskite films (Table 1) [45, 46]. The enhanced lifetime is due to the interaction of the lone pair electrons of the amino nitrogen with the uncoordinated lead ions in the perovskite film, thereby reducing the surface defects of the perovskite, which is consistent with the test results of the PL enhancement [47, 48].

**Table 1:** The values of decay amplitude constants and decay time

Film	A_1_	τ_1_ (ns)	A_2_	τ_2_ (ns)	τ_ave_ (ns)
*β-*Alanine	0.60	233	0.38	1115	896
*β-*Alanine/PEAI	0.64	370	0.27	1500	1083

In addition, the reduction in defect density by PEAI post-treatment can be verified by Mott-Schottky tests according to Eq. 1: [49]

1 $$\frac{1}{{C}^{2}}=\frac{2}{{A}^{2}e\varepsilon {\varepsilon }_{0}N}\left({V}_{\mathrm{bi}}-V\right)$$

where *C* is the measured capacitance, *A* is the active area, *V* is the bias voltage, and *N* represents carrier density. According to the calculation, the *β-*Alanine/PEAI-based PeLED (8.60 × 10^16^ V C^−2^) exhibits a larger slope than the *β-*Alanine-based PeLED (7.02 × 10^16^ V C^−2^), which indicates that *β-*Alanine/PEAI-based PeLED (7.27 × 10^15^ cm^−3^) has lower defect density than *β-*Alanine-based PeLED (8.90 × 10^15^ cm^−3^) (Fig. 4b).

Furthermore, the injection balance of electrons and holes is the key to achieve efficient PeLEDs [50–52]. In order to understand the effect of PEAI post-treatment on the charge transfer balance of PeLEDs, we tested the electron transport capability of the device with a structure of ITO/ZnO/Perovskite (270 nm)/PCBM/Ag and the hole transport capability of the device with a structure of ITO/PEDOT:PSS/Perovskite (270 nm)/Spiro-OMeTAD/Ag through single-carrier devices (Fig. 4c, d). Here the charge injection and transport behaviors are calculated by Eq. (2): [53, 54]

2 $$J = \frac{9}{8}\varepsilon_{r} \varepsilon_{o} \mu \frac{{V^{2} }}{{d^{3} }}$$

According to the calculation, the electron migration ability (*μ*_*e*_) of the *β-*Alanine and *β-*Alanine/PEAI-based perovskite films are 1.27 × 10^–3^ and 2.28 × 10^–2^ cm^2^ V^−1^ s^−1^, respectively, showing that perovskite films undergoing recrystallization allows electrons to transport more forcefully. Meanwhile, compared with the device based on *β-*Alanine-based perovskite film (2.02 × 10^–3^ cm^2^ V^−1^ s^−1^), the hole migration ability (*μ*_*h*_) of the *β-*Alanine/PEAI-based perovskite film is 2.66 × 10^–2^ cm^2^ V^−1^ s^−1^. Therefore, comparing the electron and hole migration mobility of perovskite films, it can be found that recrystallized perovskite film increases the *μ*_*e*_/*μ*_*h*_ value from 0.63 to 0.86, which ensures the effective radiative recombination of excitons in the active layer and inhibits the luminescent quenching caused by excessive carriers.

Specially, based on the reciprocal relationship between solar cell (SC) and LED, the photovoltaic performance of the devices was also tested to evaluate the nonradiative recombination loss [55, 56]. As shown in Fig. S18, without PEAI layer, the photovoltaic efficiency (PVE) of the device reaches 11.75% with a *J*_SC_ of 22.66 mA cm^−2^, a *V*_OC_ of 1.10 V, an FF of 47.12%. However, the PVE of the device with PEAI post-treatment reaches 13.76% with a *J*_SC_ of 22.87 mA cm^−2^, a *V*_OC_ of 1.14 V, and an FF of 52.79%, indicating that the device has high-performance dual functions of PV and EL. Based on the detailed balance (Shockley-Queisser, S-Q) theory, the* V*_OC_ loss caused by nonradiative recombination (∆*V*^nonrad^_OC_) is related to the EQE of LED shown in Eq. (3):

3 $$\Delta {V}_{\mathrm{OC}}^{\mathrm{nonrad}}=-\frac{kT}{q}\mathrm{lnERE}$$

where *q* the elemental charge, *k* is the Boltzmann constant, *T* is the Kelvin temperature, ∆*V*^nonrad^_OC_ is the *V*_*OC*_ loss caused by nonradiative recombination, and *ERE* is the external radiative efficiency, which is closely related to the EQE of a LED operating at the corresponding injection level. According to Eq. (3), the calculation results of ∆*V*^nonrad^_OC_ are shown in Fig. S19. The *V*_OC_ loss caused by nonradiative recombination in PeLED decreased from 56 to 40 meV after PEAI post-treatment, indicating that the nonradiative recombination was effectively inhibited, which was also consistent with the *V*_OC_ of PVE, and proved the reciprocal relationship was established.

To gain more insight into the effect of PEAI post-treatment on the carrier recombination kinetics, we also measured the light intensity dependent *J-V* curves. Figure S20 shows the curves of *J*_SC_ (*J*_*SC*_ ∝ I^α^) and *V*_OC_ values with light intensity. The ideal factor (*n*_*2*_) in *n*_*2*_*k*_B_*T/q* is determined by the slope of *V*_OC_ relative to the light intensity in the semi-logarithmic scale, which can be expressed by Eq. 4 [57]:

4 $${V}_{\mathrm{OC}}=\frac{n{k}_{\mathrm{B}}T}{q}\mathrm{ln}(P)$$

where *k*_*B*_ is Boltzmann constant, *T* is absolute temperature, *P* is light intensity, and* n* is ideality factor. As shown in Fig. S20a, compared with the slope of *β-*Alanine-based device (1.86 *k*_B_*T/q*), the slope of the *β-*Alanine/PEAI-based device is decreased to 1.58 *k*_B_*T/q*, showing that the major trap-assisted recombination was effectively inhibited in device [58]. In addition, compared with the *β-*Alanine-based device (α = 0.93), the *α* value of the *β-*Alanine/PEAI-based device is 0.97, indicating that the *β-*Alanine/PEAI-based device can effectively balance charge transfer (Fig. S20b). Therefore, PEAI post-treatment can not only balance the charge transfer but also suppress the defect state of perovskite film and effectively reduce nonradiative recombination, thus improving the multifunctional performance of the device.

## Conclusions

In this work, we adopted post-treatment strategy to induce downward recrystallization for spontaneous formation of buried submicrocavities as light output coupler. Based on the calculation, the LOCE of the device is increased from 26.8 to 36.2%. Beyond that, the perovskite film after PEAI post-treatment can better match the energy levels with the interfacial layers and reduce the carrier injection barrier, which provides advantages for the fabrication of highly efficient PeLEDs. Therefore, our PeLED achieved an unprecedented EQE of 25.5% with a radiance of 487 W sr^−1^ m^−2^ and low rolling-off. The turn-on voltage decreased from 1.25 to 1.15 V at 0.1 W sr^−1^ m^−2^. The *T*_75_ stability exceeds 15 h at a current density of 62.5 mA cm^−2^. This work provides a self-assembly method to integrate output coupler for boosting the performance of PeLEDs.

### Supplementary Information

Below is the link to the electronic supplementary material.

Supplementary file1 (PDF 6591 KB)
