# Supplementary material for: Self-Generated Buried Submicrocavities for High-Performance Near-Infrared Perovskite Light-Emitting Diode
Source: Nanomicro Lett. 2023 May 15;15:125. doi: 10.1007/s40820-023-01097-3 (PMC10185725; doi:10.1007/s40820-023-01097-3)
Supplement: Supplementary file 1 — Supplementary file1 (PDF 6591 KB) [file 40820_2023_1097_MOESM1_ESM.pdf]

Supporting Information for

Self-Generated Buried Submicrocavities for High Performance Near-Infrared Perovskite Light-Emitting Diode

Jiong Li[1], Chenghao Duan[1, 3], Qianpeng Zhang[2], Chang Chen[1], Qiaoyun Wen[1], Minchao Qin[3], Christopher C. S. Chan[4], Shibing Zou[1], Jianwu Wei[5], Zuo Xiao[6], Chuantian Zuo[6], Xinhui Lu[3], Kam Sing Wong[4], Zhiyong Fan[2, *], Keyou Yan[1, *]

[1]School of Environment and Energy, State Key Lab of Luminescent Materials and Devices, Guangdong Provincial Key Laboratory of Solid Wastes Pollution Control and Recycling, South China University of Technology, Guangzhou 510000, P. R. China

[2]Department of Electronic and Computer Engineering, The Hong Kong University of Science and Technology, Clear Water Bay, Kowloon, Hong Kong SAR, P. R. China.

[3]Department of Physics, The Chinese University of Hong Kong, Shatin, Hong Kong, P. R. China

[4]Department of Physics, The Hong Kong University of Science and Technology, Clear Water Bay, Kowloon, Hong Kong 999077, P. R. China.

[5]School of Chemistry and Chemical Engineering, Guangxi University, Nanning 530004, P. R. China

[6]Center for Excellence in Nanoscience (CAS), Key Laboratory of Nanosystem and Hierarchical Fabrication (CAS), National Center for Nanoscience and Technology, Beijing 100190, P. R. China

*Corresponding authors. E-mail: kyyan@scut.edu.cn (Keyou Yan), eezfan@ust.hk (Zhiyong Fan)

Supplementary Figures

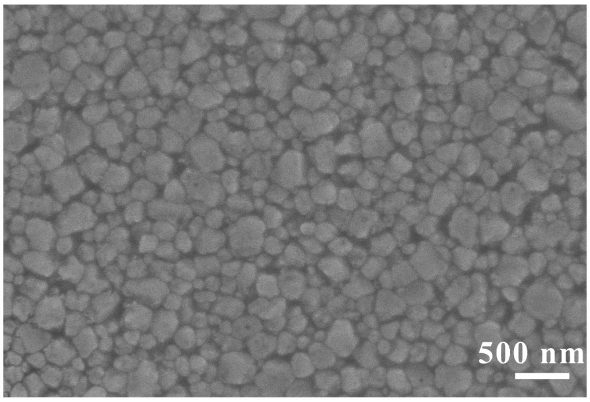

Fig. S1 SEM image of perovskite film (FAI: PbI_2 = 1.8:1)

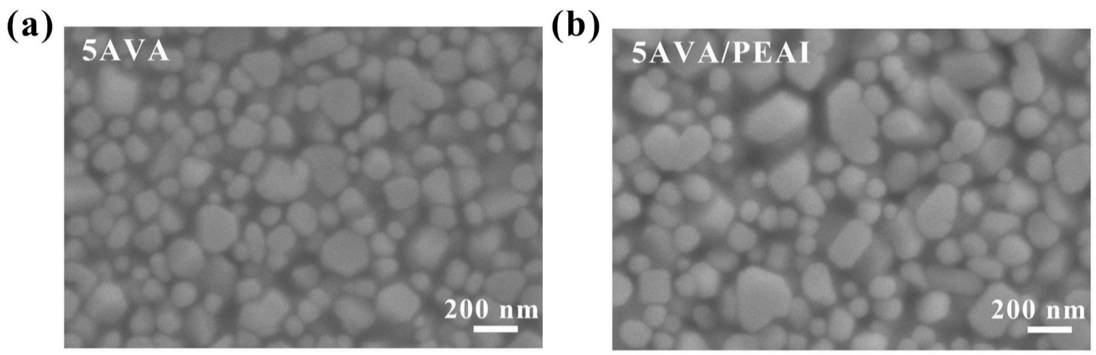

Fig. S2 SEM images of perovskite films. **a** 5AVA. **b** 5AVA/PEAI

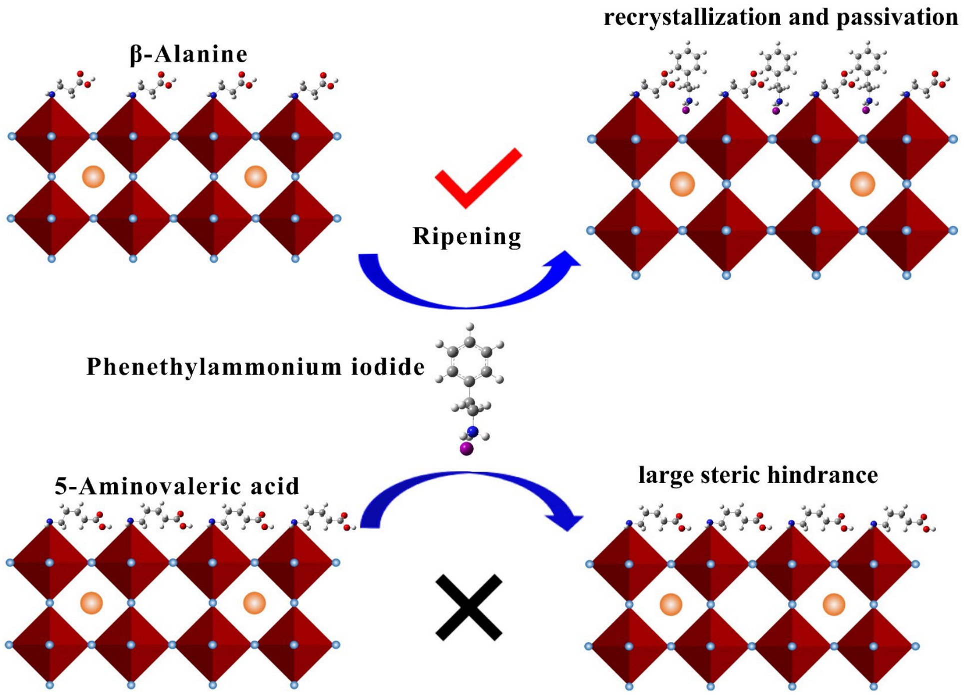

Fig. S3 Schematic diagram of PEAI post-processing

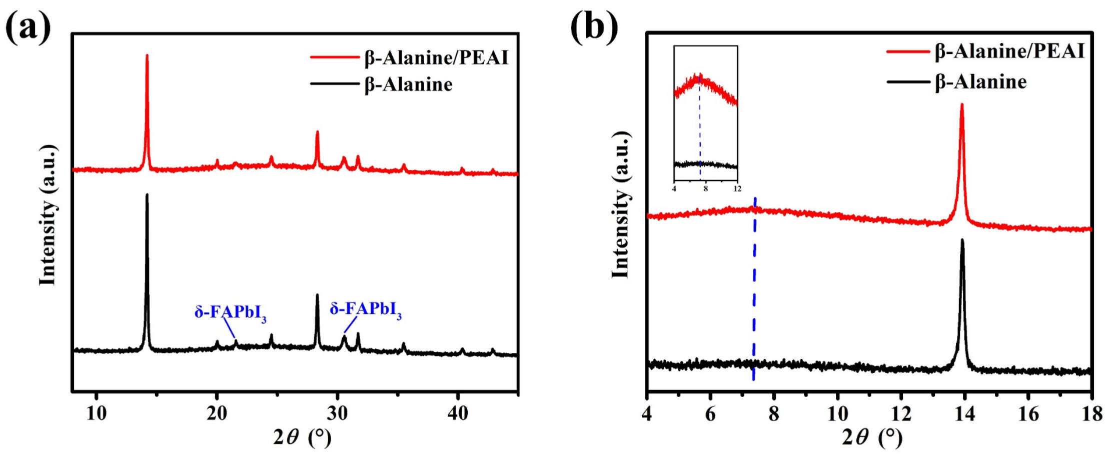

Fig. S4 XRD patterns of the perovskite films

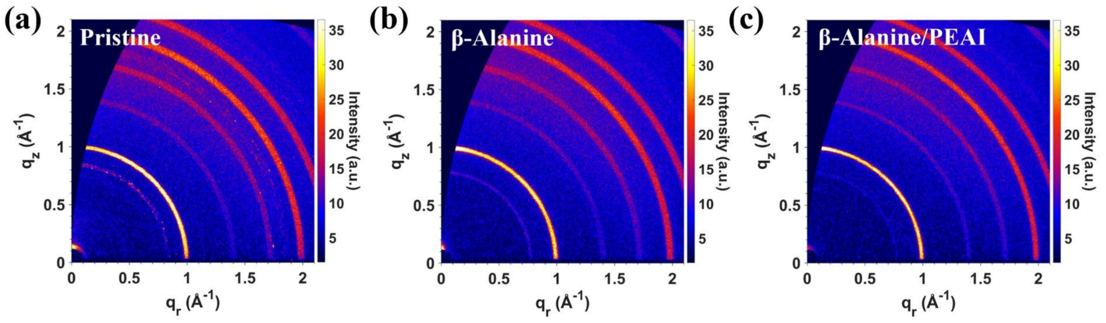

Fig. S5 2D GIWAXS patterns of perovskite films. **a** Pristine. **b** β-Alanine. **c** β-Alanine/PEAI

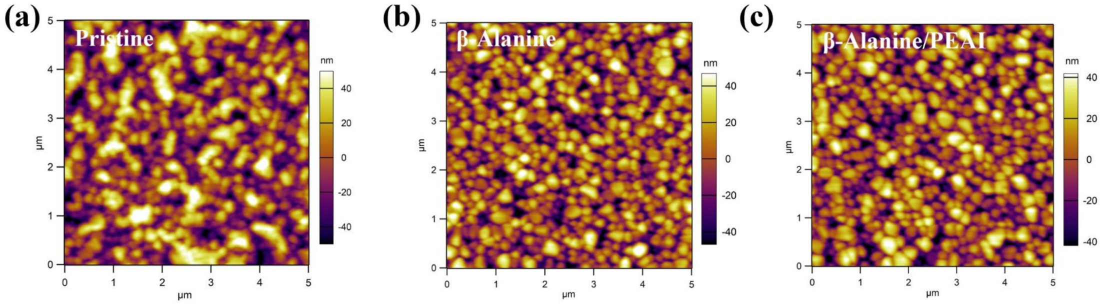

Fig. S6 AFM measurements of perovskite films. **a** Pristine. **b** β-Alanine. **c** β-Alanine/PEAI

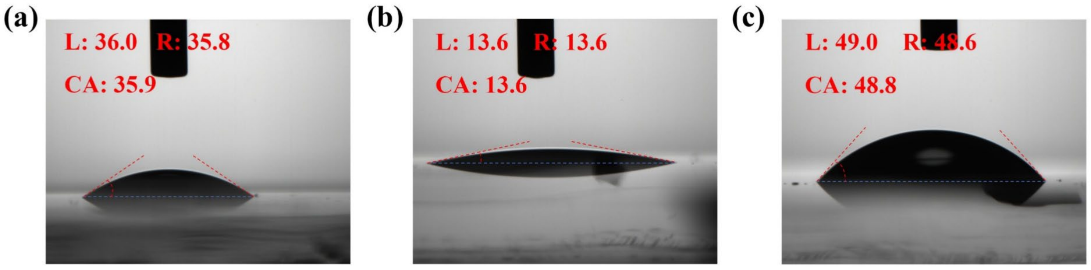

Fig. S7 The water contacts angle measurements of perovskite films. **a** Pristine. **b** β-Alanine. **c** β-Alanine/PEAI

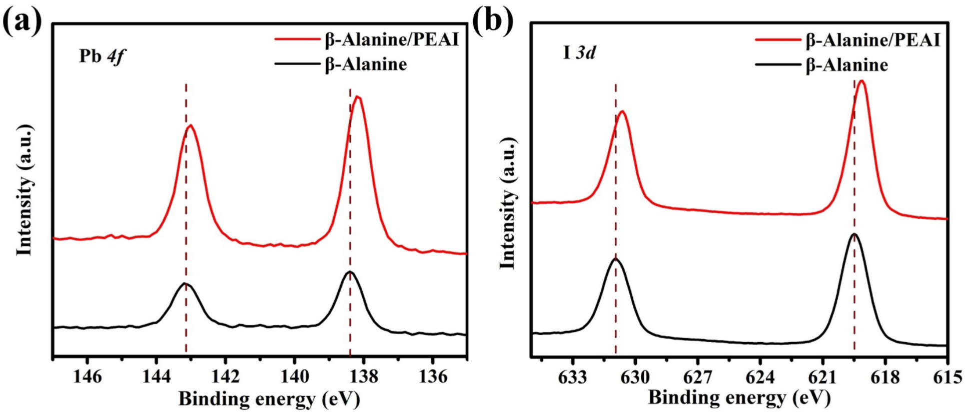

Fig. S8 XPS spectra of perovskite films. **a** Pb *4f* peak. **b** I *3d* peak

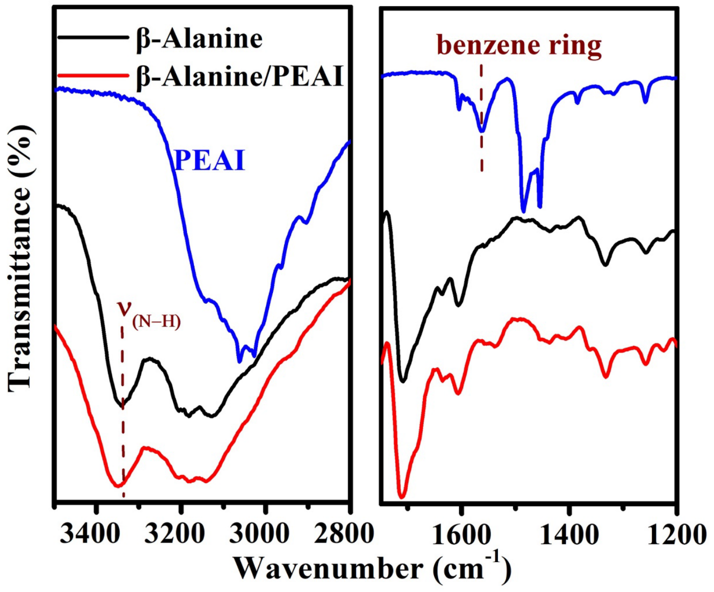

Fig. S9 FTIR spectra of perovskite films

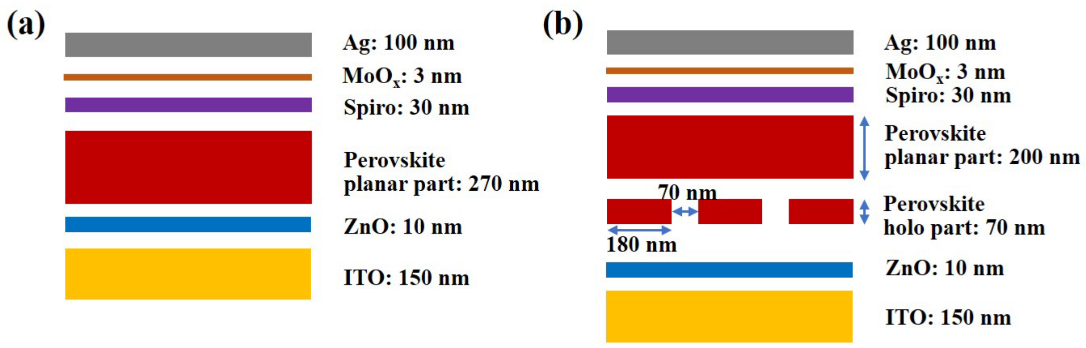

Fig. S10 Simulation models. **a** Planar-structure. **b** Holo-structure

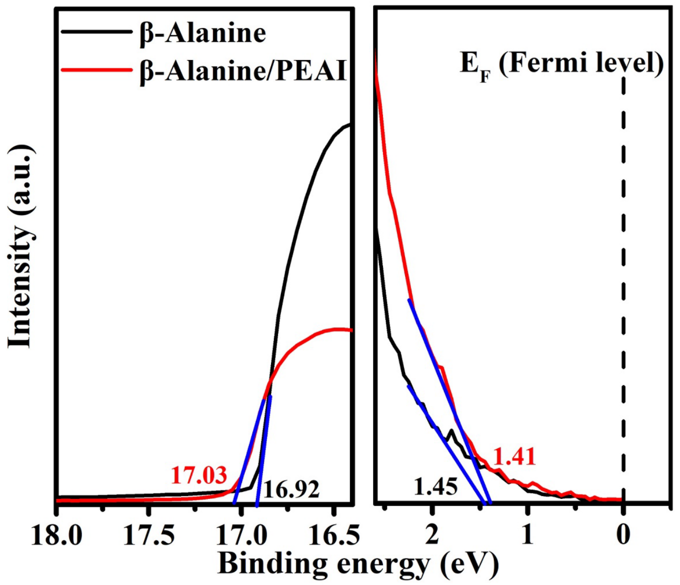

Fig. S11 UPS spectra of perovskite films

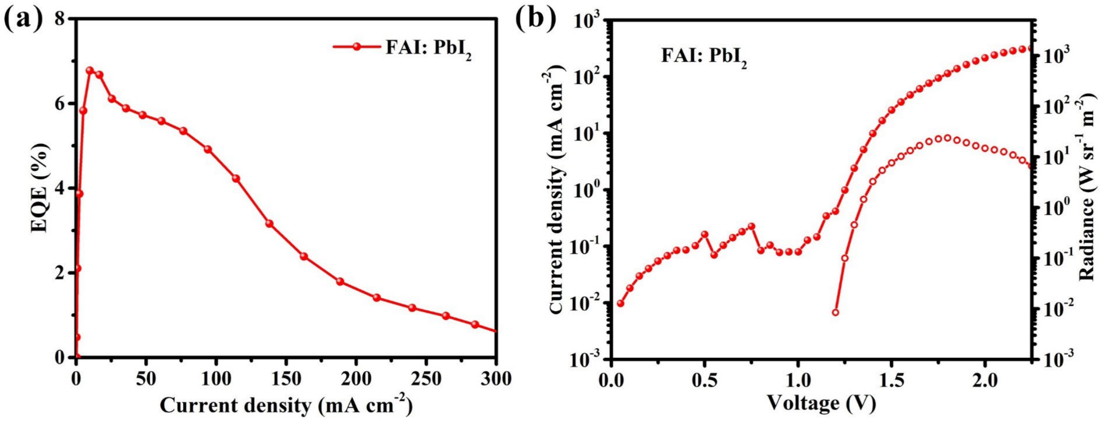

Fig. S12 a EQE diagram of pristine PeLED. **b** The Current density-voltage-radiance curves of pristine PeLED

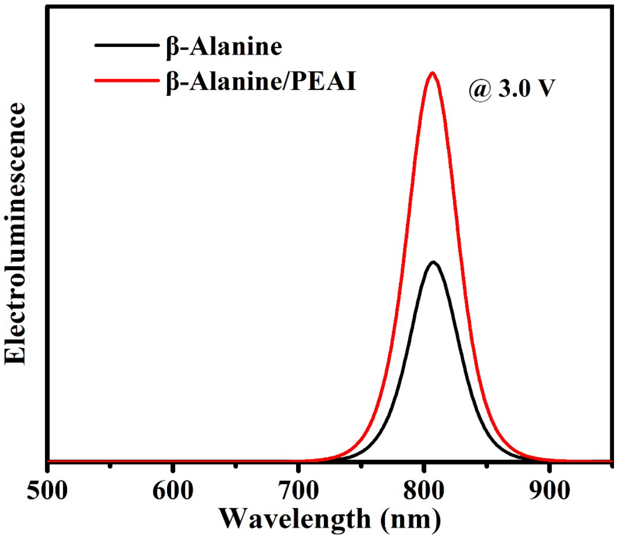

Fig. S13 EL spectra of the PeLEDs without and with PEAI post-treatment

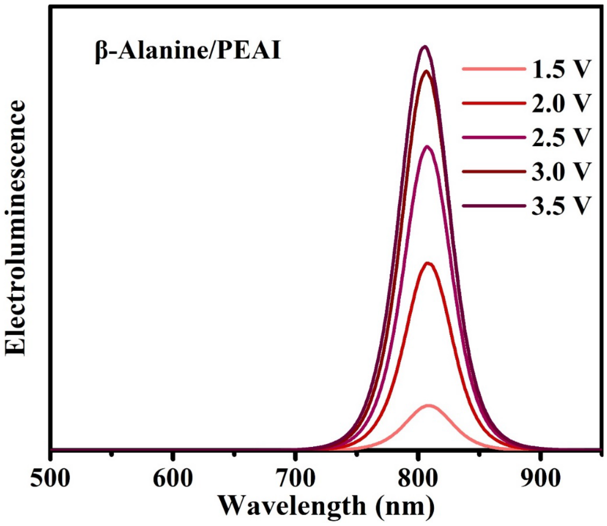

Fig. S14 EL spectra of the PeLED with PEAI post-treatment under different bias voltages

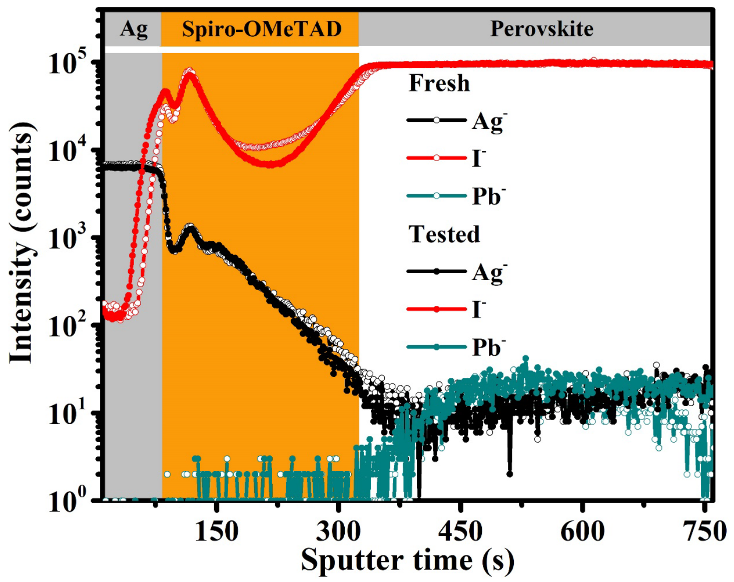

Fig. S15 ToF-SIMS of depth profiles of anion distribution within full PeLED

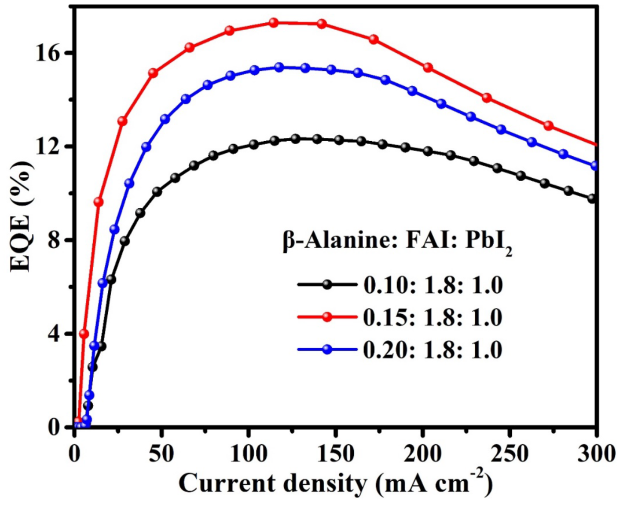

Fig. S16 The effect of β-Alanine concentration on EQE of PeLEDs

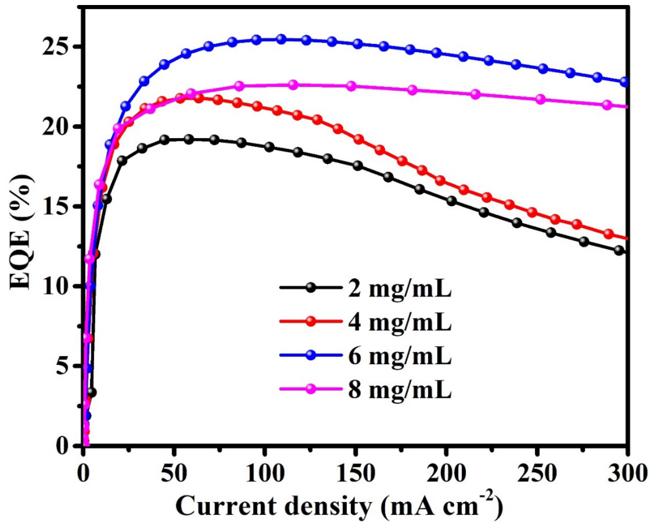

Fig. S17 The effect of PEAI concentration on EQE of PeLEDs

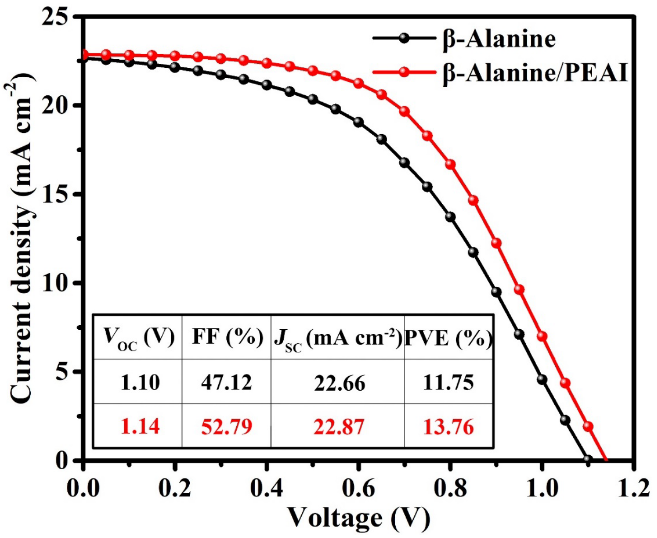

Fig. S18 *J-V* characteristic curves of the PeLEDs

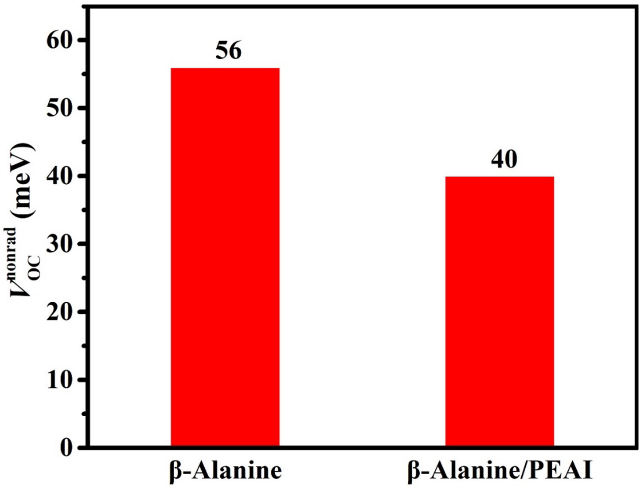

Fig. S19 V_{OC} losses caused by nonradiative recombination of devices without and with PEAI post-treatment

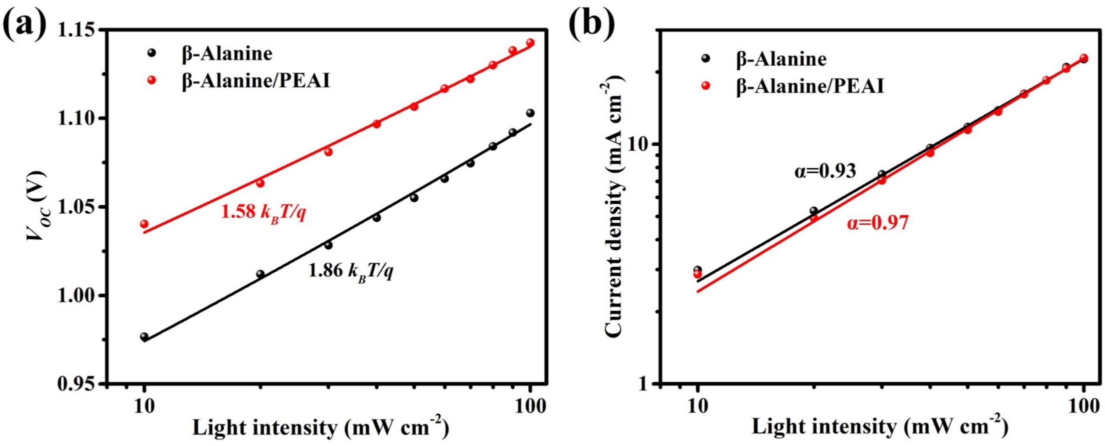

Fig. S20 a The changes of V_{OC} against light intensity of the devices. **b** The changes of current density against light intensity of the devices
